# Toward Single Bacterium Proteomics

**DOI:** 10.1021/jasms.3c00242

**Published:** 2023-09-15

**Authors:** Ákos Végvári, Xuepei Zhang, Roman A. Zubarev

**Affiliations:** Division of Chemistry I, Department of Medical Biochemistry and Biophysics, Karolinska Institutet, SE-171 77 Stockholm, Sweden

**Keywords:** Escherichia coli, bacterial proteome, single cell proteomics

## Abstract

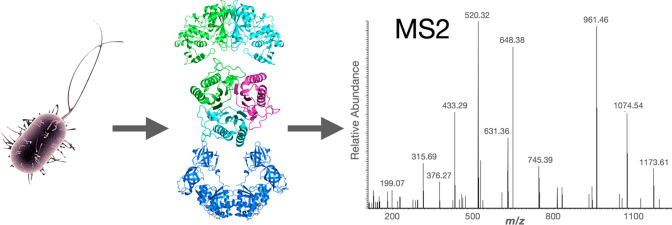

Bacteria are orders of magnitude smaller than mammalian cells, and while single cell proteomics (SCP) currently detects and quantifies several thousands of proteins per mammalian cell, it is not clear whether conventional SCP methods will be suitable for bacteria. Here we report on the first successful attempt to detect proteins from individual *Escherichia coli* bacteria, with validation of our findings by comparison with two bacteria samples and bulk proteomics data. Data are available via ProteomeXchange with the identifier PXD043473.

## Introduction

Single Cell ProtEomics by Mass Spectrometry (SCoPE-MS) by Budnik and Slavov^1^ revolutionized in 2017–2018 the nascent field of single cell proteomics (SCP), opening a venue for innovative approaches in ultrasensitive sample preparation and mass spectrometry analysis. The key innovation in SCoPE MS was the use of the carrier proteome (CP) composed of 100–200 cells and multiplexed with single cell samples using isobaric tandem mass tag (TMT). With SCoPE-MS, it is habitually possible to analyze human cells to the depth of 1500–2000 proteins.^2,3^ There are dozens of recent reports with similar or greater proteome analysis depths owing to continuous advances in sample preparation,^4−8^ data acquisition expanding the choice of analytical approaches to label-free technique and with data independent acquisition modes,^4,9−12^ as well as providing optimal data analysis.^13,14^

The next challenge in single cell proteomics would be to detect proteins from single bacteria. Bacteria come in a wide range of sizes, from the smallest (*Mycoplasma gallicepticum w*ith a size of 0.2–0.3 μm) to the largest (a Gram-negative proteobacterium *Thiomargarita namibiensis*, up to 750 μm).^15^ Gram-negative *Escherichia coli* bacteria that are most commonly used in research are much smaller than human cells, but they are easy to grow and easier to lyse than Gram-positive bacteria. The typical diameter of A549 human cell is 11–15 μm,^16^ with a volume of ≈1000 μm^3^, while *E. coli* cells represent a cylinder of 1.0–2.0 μm long with a radius of about 0.5 μm and a volume of ≈1 μm^3^.^17^ Other sources give different values for *E. coli* size ranges, *e.g*., 2–6 μm long and 1.1–1.5 μm wide.^18^ This discrepancy is due to the fact that the size and volume of the bacterial cell depend upon the growth rate. In general, faster dividing cells are larger than slower growing bacteria. The dry protein mass of *E. coli* cells varies from an average value of 148 fg per cell for bacteria dividing every 100 min to 865 fg per cell for those with a 24 min division time, a >5-fold difference.^19^

The proteome of *E. coli* contains about 4200 proteins.^20^ Some of the most abundant proteins in the *E. coli* proteome are ribosomal proteins. The number of ribosomes is steeply dependent on the growth rate: as one would expect, the faster the growth rate, the more ribosomes that are present.^21^ It is believed that, under optimal growing conditions, a single *E. coli* bacterium may have up to 60,000 ribosomes. Assuming such a value, it should be possible to detect and quantify at least most abundant ribosomal proteins (the L7/12 complex is present in four copies per ribosome) by SCoPE-MS, although soluble enzymes (>1000) are also potential target of such analyses. In order to validate protein detection, we compared single bacterium proteomics (SBP) results with the bulk proteome analysis of bacteria, demonstrating that single cell analysis identified proteins that are very abundant in bulk proteomics data.

## Materials and Methods

### Culturing Bacterial Cells

*Escherichia coli* BL21(DE3) strain was grown on plates containing LB agar (Sigma), and a single colony was transferred to Terrific Broth (TB) medium. Bacterial growth was monitored by measuring light diffraction at 600 nm on a BioScreen C automated microbiology growth curve analysis system (Bioscreen, Finland) at 37 °C. After 4 h, the bacteria were washed with PBS twice and incubated in PBS containing 5 mM SYTO 9 green fluorescent nucleic acid stain (Thermo Fisher Scientific, S34854) at room temperature (RT) for 15 min. The remaining SYTO 9 dye was removed by centrifugation, and the bacteria were washed with PBS.

### Sample Preparation

For bulk proteome analysis, the harvested and washed cells were lysed with two different methods following supplementation with either 100 μL of 25 mM triethylammonuim bicarbonate (TEAB) for probe sonication method or 50 μL of water for the freeze-and-thaw method and sonicated in water bath for 10 min. The cells either were sonicated twice using an ultrasound probe (Vibracell) for 1 min operated with a pulse of 2–2 s (on–off) and amplitude to 20% after or underwent four freeze-and-thaw cycles, being frozen in liquid N_2_ and then heated on a block heater at 70 °C for 2 min in each cycle. Supernatants were used for determination of protein concentration by the BCA method. Thereafter 25 μg of cell extract was supplemented with 50 mM TEAB to reach a total volume of 100 μL, and proteins were digested without reduction and alkylation by adding 10 μL of 0.1 μg/μL sequencing grade trypsin (Promega) and incubating at 37 °C for ca. 16 h. The resulting peptides were labeled with 500 μg of TMT-10plex (Thermo Fisher Scientific) in 150 μL of dry acetonitrile (ACN) by incubating at RT for 2 h with gentle shaking. The reaction was quenched by adding 15 μL of 5% hydroxylamine (Sigma). The peptides labeled with different TMT labels were pooled together and dried in a vacuum concentrator (Eppendorf).

For single cell proteomics, bacteria were stained with SYTO 9 to facilitate sorting to a 96-well plate in an BD FACSAria III flow cytometer system (BD Biosciences) either one or two cells into wells holding predispensed 5 μL of 100 mM TEAB. In addition, 250 cells to be used as CP were sorted into some wells. As described previously,^3^ proteins were extracted by four freeze-and-thaw cycles using liquid nitrogen and incubation at 70 °C for 2 min in each cycle with a 2 min interval at RT between each cycle. Proteins were denatured by heating the plates to 90 °C for 10 min. Then 1 or 2 μL of sequencing grade trypsin (Promega) in 50 mM TEAB was added to single/double cells and CP, respectively, and incubated at 37 °C for 16 h. Labeling with TMT-10plex was performed with dispensing in each well 1 μL of a 10 ng/μL reagent solution using a MANTIS liquid handling robot (Formulatrix) and incubating at RT for 1 h with gentle shaking and subsequent quenching for 20 min at RT with 1 μL of 5% hydrocylamine. Labeled peptides from three single and three double cells were pooled together with two CP digests labeled by two different TMT labels and dried in vacuum prior to analysis. Two TMT channels remained unutilized to determine the background noise level.

### RPLC-LC-MS/MS Analysis

LC-MS/MS analysis of the bulk proteome sample was performed on an Ultimate 3000 UPLC coupled to a Q Exactive HF hybrid mass spectrometer (Thermo Fisher Scientific). Peptides were loaded onto a trap column (Acclaim PepMap 100 precolumn, 75 μm diameter, 2 cm long, 3 μm C18 beads, 100 Å pores, Thermo Scientific) and separated on an EASY-Spray analytical column (50 cm long, 75 μm i.d., PepMap RSLC C18 2 μm beads, 100 Å pores, Thermo Scientific) using a 120 min long linear gradient from 4% to 26% solvent B (0.1% FA in 98% ACN and 2% water) at a flow rate of 300 nL/min and a column temperature of 55 °C. Mass spectrometry (MS) acquisition method was set as follows: full MS spectra at *m*/*z* 375–1700 with a resolution of 120,000 (at 200 *m*/*z*), with an automated gain control (AGC) target value of 1 × 10^6^ and 80 ms injection time (IT), with data dependent acquisition (DDA) selection for fragmentation of 18 most abundant ions. MS/MS was performed with an isolation window of 1.4 Th and fragmentation by higher-energy collision dissociation (HCD) at a normalized collision energy (NCE) of 34%, maximum IT of 54 ms, and AGC target of 2 × 10^5^. Fragment ion detection was in the Orbitrap HD analyzer at a resolution of 60,000 with fixed first mass at 110 *m*/*z* and with a dynamic exclusion of 45 s.

LC-MS/MS analysis of single cells was performed with a nanoflow UltiMate 3000 UPLC coupled to an Orbitrap Fusion Eclipse Tribrid mass spectrometer equipped with field asymmetric ion mobility device FAIMS Pro (Thermo Fisher Scientific). The peptides were separated on a 25 cm Easy-Spray PepMap C-18 column (Thermo Fisher Scientific) using a gradient from 1%B to 20%B in 75 min and from 1%B to 36%B in 20 min at a 300 nL/min flow rate. FAIMS operated at a compensation voltage cycling every 1.5 s between −50 and −70 V. The mass spectrometer was used in the DDA mode. The precursor ions were recorded at 120,000 resolution in the *m*/*z* 250–1500 range, targeting 6 × 10^5^ ions in maximum 100 ms, while MS/MS precursors were isolated with a AGC target of 1 × 10^5^ in maximum 50 ms with a narrow 0.7 Th window and fragmented by HCD at 35% NCE. The fragment ions were detected at 50,000 resolution with dynamic exclusion of 60 s.

Peptides and proteins were identified by searching MS/MS data against the *E. coli* strain K12 protein database (SwissProt, 4465 entries) by MS Amanda^22^ using Proteome Discoverer 2.5 software. The precursor and fragment ion mass tolerances were 10 ppm and 0.05 Da, respectively. Variable modifications were set for deamidation of asparagine and glutamine, oxidation of methionine, and “TMT6plex” (+229.163 Da; same as TMT10plex) of lysine and peptide N-termini. The protein list was filtered with an estimated false discovery rate (FDR) of 5% and validation based on *q*-values using either the Percolator or Target Decoy PSM validator node in Proteome Discoverer. The protein group abundances were calculated as a sum of the peptide abundances determined in Proteome Discoverer (Reporter Ion Quantifier node) based on the detected levels of TMT reporter ions.

The mass spectrometry proteomics data have been deposited to the ProteomeXchange Consortium via the PRIDE^23^ partner repository with the data set identifier PXD043473.

## Results and Discussion

### Bulk Proteomics

In total, 1132 and 1270 bacterial proteins were detected and quantified in bulk analysis across three replicates prepared with freeze-and-thaw and probe sonication, respectively. As we were interested in the most abundant proteins, there was no need to reach a deeper proteome. Among the detected proteins, there were 34 molecules localized in the small subunit of the ribosome and 43 proteins from the large ribosome subunit; eight additional ribosome related proteins were detected. Overall, ribosomal proteins comprise 12.1% of the total protein abundance.

**Figure 1:**
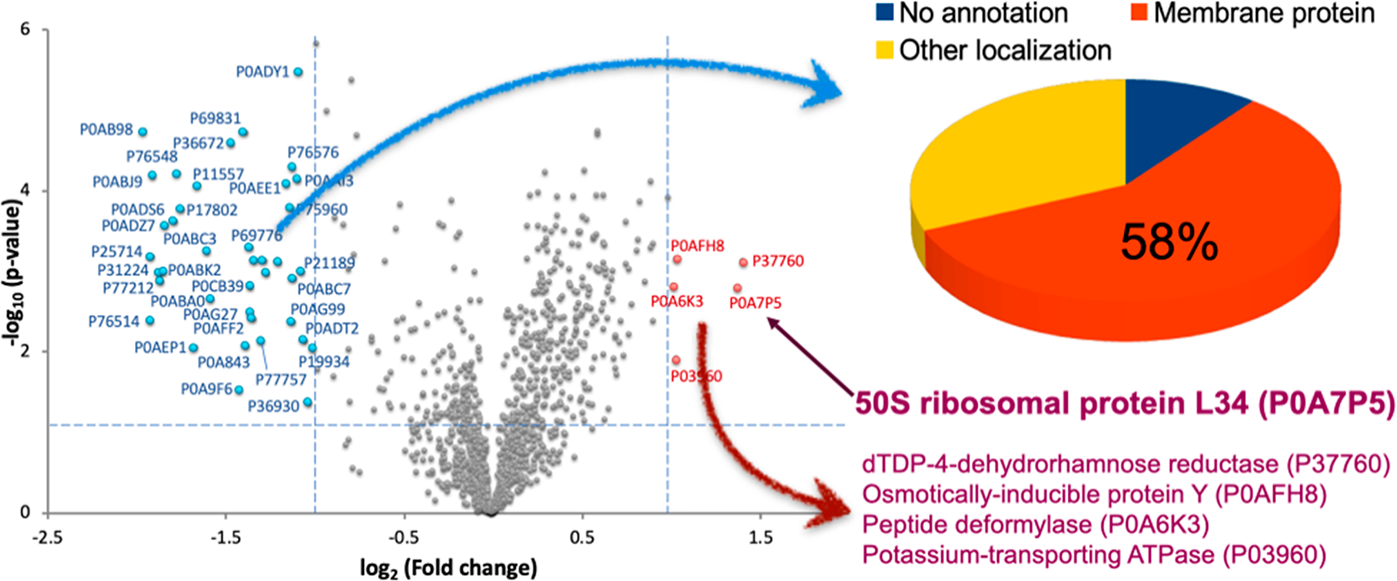
Comparison of sample preparation methods in bulk proteomics. The freeze-and-thaw method extracted 53% more proteins than the probe sonication method did, including a ribosomal protein (L34). The less effectively extracted proteins included mostly membrane proteins (58%) as depicted in the pie chart.

The comparison of the two preparation methods indicated significant differences in extraction efficiency for some protein groups (Figure 1). Probe sonication processed membrane related proteins more effectively, as about 58% of proteins significantly enriched in this approach fell in this category. However, the freeze-and-thaw method provided a higher yield of more than half of the detected proteins, and importantly, one significantly enriched protein was ribosomal (P0A7P5, 50S ribosomal protein L34). Therefore, we decided that the freeze-and-thaw approach is suitable for single bacterium proteomics (SBP) analysis.

### Single Bacterium Proteomics

**Figure 2:**
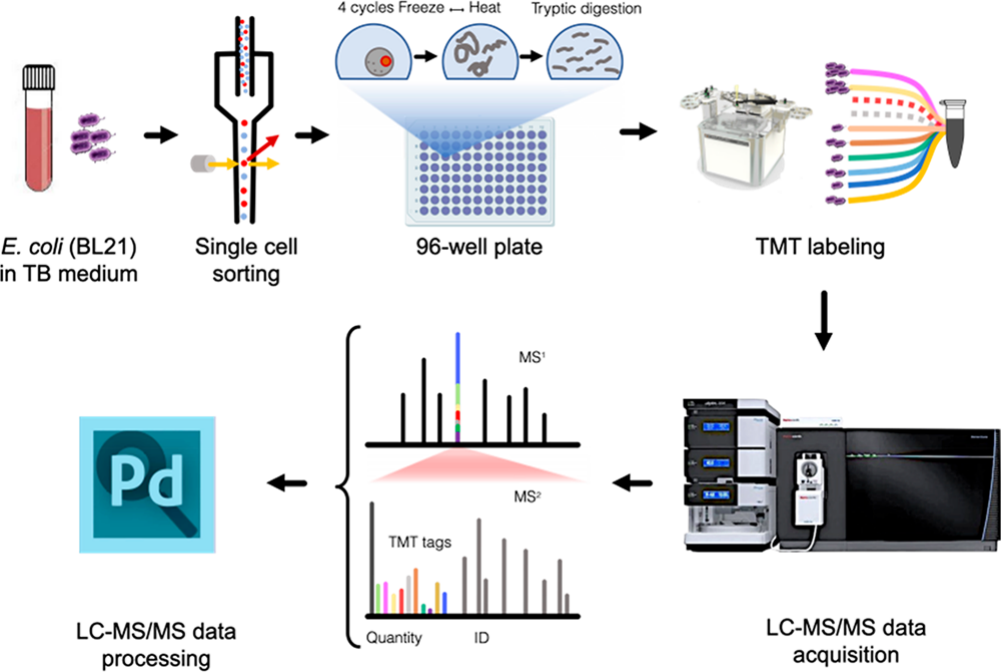
Single bacterium proteomics workflow. The cultured *E. coli* cells were isolated as single or double cells on 96-well plates by FACS; lysed by freeze-and-thaw, and digested before TMT-10plex labeling. Data acquisition was achieved on an Orbitrap Fusion Eclipse mass spectrometer equipped with FAIMS Pro, collecting MS2 spectra for reporter ion based quantification.

The overall SBP workflow is presented in Figure 2. Each TMT-10 multiplexed set contained two TMT-channels with 250 bacterial cells each as CP, as well as three single cell and three double cell channels in alternating order. In total, 16 such TMT sets were analyzed. Altogether, 388,641 MS/MS spectra were collected resulting in over 20,000 peptide-spectrum matches (PSMs), among which many mapped on common contaminant proteins (e.g., keratins) as well as trypsin. The TMT labeling efficiency was determined to be 75%, which was somewhat lower than the average labeling efficiency of 85% in our previous work with single mammalian cells,^3^ but still sufficient for the purpose of this work.

Using Percolator for control of FDR ≤ 0.05, 94 PSMs were annotated in the *E. coli* database, identifying 29 peptides of 19 bacterial proteins across the 96 single/double cells (Tables 1 and 2). The following three proteins were identified with ≥2 peptides each: elongation factor Tu 2 (P0CE48), glyceraldehyde-3 phosphate dehydrogenase 3 A (P0A9B2), and 2-iminobutanoate/2-iminopropanoate deaminase (P0AF93).

**Table 1:** Summary of Detected Bacterial Proteins and Peptides Determined by the Percolator Method, Including Channel Occupancy Values Based on the Observation Frequencies in Single, Double, and Carrier Proteome Samples^a^

protein description	gene name	accession number [position]	annotated sequence	theoretical [MH]^+^ (Da)	observed *m*/*z* (Da)	charge state (z)	retention time (min)	# PSM	protein score (by MS Amanda)	channel occupancy (%)	abundance rank in bulk proteome
tRNA (guanine-N(1)-)-methyltransferase	trmD	P0A873 [69–75]	[R].DAIHAAK.[A]	1183.72	395.25	+3	31.52	3	214.27	18.75%	
glyceraldehyde-3-phosphate dehydrogenase A	gapA	P0A9B2 [218–225]	[K].VLPELNGK.[L]	1328.82	664.92	+2	69.00	8	221.57	50.00%	1
2-iminobutanoate/2-iminopropanoate deaminase	ridA	P0AF93 [52–58]	[R].QSLDNVK.[A]	1033.57	517.29	+2	36.19	3	342.48	18.75%	242
major outer membrane lipoprotein Lpp	lpp	P69776 [53–59]	[R].SDVQAAK.[D]	1176.70	588.85	+2	33.77	4	190.35	25.00%	466
*N*-acetylneuraminate lyase	nanA	P0A6L4 [242–248]	[K].VIDLLIK.[T]	1271.87	636.44	+2	86.04	4	205.75	25.00%	
50S ribosomal protein L7/L12	rplL	P0A7K2 [110–121]	[K].ALEEAGAEVEVK.[-]	1702.96	568.33	+3	63.17	16	252.42	93.75%	21
elongation factor Tu 2	tufB	P0CE48 [239–249]	[K].VGEEVEIVGIK.[E]	1629.98	544.00	+3	75.80	15	203.77	75.00%	11
biotin carboxylase	accC	P24182 [315–324]	[R].IAAGQPLSIK.[Q]	1456.91	728.96	+2	71.29	2	147.8	12.50%	120
probable cyclic di-GMP phosphodiesterase PdeC	pdeC	P32701 [36–43]	[K].SEVNNQLR.[T]	1189.64	595.33	+2	41.77	4	177.29	25.00%	
succinylornithine transaminase	astC	P77581 [353–360]	[K].QISQEAAK.[A]	1333.77	667.39	+2	45.17	1	189.61	6.25%	
elongation factor Tu 2	tufB	P0CE48 [178–188]	[K].ALEGDAEWEAK.[I]	1676.89	559.64	+3	63.80	10	127.64	50.00%	11
2-iminobutanoate/2-iminopropanoate deaminase	ridA	P0AF93 [52–58]	[R].QSLDNVK.[A]	1262.74	631.87	+2	44.89	1	186.3	6.25%	242
ATP-dependent RNA helicase HrpA	hrpA	P43329 [1049–1063]	[R].DSVAIKLFDNPLEQK.[Q]	2176.23	726.08	+3	91.39	7	104.31	25.00%	
putative permease PerM	perM	P0AFI9 [179–188]	[K].DKEQMLNAVR.[R]	1450.74	725.88	+2	53.56	4	172.05	18.75%	
4-hydroxybenzoate octaprenyltransferase	ubiA	P0AGK1 [61–79]	[R].AAGCVVNDYADRKFDGHVK.[R]	2294.15	765.39	+3	66.52	5	194.66	12.50%	
*uncharacterized protein YdaV*	*ydaV*	*P77546[180–197]*	*[K].NEQVVLHQIVDRRTASMR.[S]*	*2171.09*	*724.37*	*+3*	*81.32*	*11*	*160.15*	*0.00%*	
*outer membrane usher protein HtrE*	*htrE*	*P33129[148–159]*	*[R].LDIDVPQAWVMK.[N]*	*1643.90*	*548.64*	*+3*	*52.73*	*1*	*136.87*	*0.00%*	
*elongation factor* Tu 2	*tufB*	*P0CE48* [*189–205]*	*[K].ILELAGFLDSYIPEPER.[A]*	*2191.18*	*731.07*	*+3*	*97.60*	*1*	*120.2*	*0.00%*	
*elongation factor* Tu 2	*tufB*	*P0CE48[125–137]*	*[R].QVGVPYIIVFLNK.[C]*	*1948.20*	*650.07*	*+3*	*92.11*	*5*	*118.38*	*0.00%*	
*methylated-DNA-protein-cysteine methyltransferase*	*ogt*	*P0AFH0[54–67]*	*[R].ISATNPGGLSDKLR.[E]*	*1428.78*	*714.90*	*+2*	*65.42*	*1*	*138.33*	*0.00%*	*1081*
*ATP synthase subunit alpha*	*atpA*	*P0ABB0[165–175]*	*[R].ELIIGDRQTGK.[T]*	*1229.68*	*615.35*	*+2*	*30.30*	*1*	*228.28*	*0.00%*	*33*
*glyceraldehyde-3-phosphate* dehydrogenase A	*gapA*	*P0A9B2[214–225]*	*[K].AVGKVLPELNGK.[L]*	*1224.73*	*408.92*	*+3*	*39.82*	*1*	*191.31*	*0.00%*	*1*
*glyceraldehyde-3-phosphate* dehydrogenase A	*gapA*	*P0A9B2[226–232]*	*[K].LTGMAFR.[V]*	*795.42*	*398.22*	*+2*	*43.10*	*3*	*179.87*	*0.00%*	*1*
*glyceraldehyde-3-phosphate* dehydrogenase A	*gapA*	*P0A9B2[5–12]*	*[K].VGINGFGR.[I]*	*819.45*	*410.23*	*+2*	*41.50*	*1*	*173.49*	*0.00%*	*1*
*glyceraldehyde-3-phosphate* dehydrogenase A	*gapA*	*P0A9B2[218–232]*	*[K].VLPELNGKLTGMAFR.[V]*	*1645.91*	*549.31*	*+3*	*68.35*	*2*	*191.49*	*0.00%*	*1*
*glyceraldehyde-3-phosphate* dehydrogenase A	*gapA*	*P0A9B2[218–232]*	*[K].VLPELNGKLTGMAFR.[V]*	*1661.90*	*554.64*	*+3*	*60.08*	*1*	*395.37*	*0.00%*	*1*
*glyceraldehyde-3-phosphate* dehydrogenase A	*gapA*	*P0A9B2[218–232]*	*[K].VLPELNGKLTGMAFR.[V]*	*1662.89*	*554.97*	*+3*	*63.85*	*1*	*237.95*	*0.00%*	*1*
transaldolase A	*talA*	*P0A867[92–99]*	*[R].VSTEVDAR.[L]*	*876.44*	*438.73*	*+2*	*19.09*	*2*	*246.62*	*0.00%*	*976*
*chaperone protein DnaK*	*dnaK*	*P0A6Y8[167–183]*	*[K].RIINEPTAAALAYGLDK.[G]*	*1816.00*	*606.01*	*+3*	*64.08*	*1*	*285.59*	*0.00%*	*15*

a Italic = observation only without quantification.

**Table 2:** Summary of Detected Bacterial Proteins and Peptides Determined by the Targeted Decoy Method, Including Channel Occupancy Values Based on the Observation Frequencies in Single, Double, and Carrier Proteome Samples^a^

protein description	gene name	accession number [position]	annotated sequence	theoretical [MH]^+^ (Da)	observed *m*/*z* (Da)	charge state (z)	retention time (min)	# PSM	protein score (by MS Amanda)	channel occupancy (%)	abundance rank in bulk proteome
protein CbrA	cbrA	P31456 [22–33]	[K].LAGKMQVIALDK.[K]	1761.07	587.69	+3	89.87	4	228.5	25.00%	
HTH-type transcriptional regulator GadX	gadX	P37639 [243–250]	[R].SAQRLSNR.[D]	1161.65	581.33	+2	30.74	2	191.41	12.50%	
ribomal silencing factor RsfS	rsfS	P0AAT6 [1–13]	[-].MQGKALQDFVIDK.[I]	1952.09	651.36	+3	45.44	1	185.02	6.25%	573
uncharacterized protein YbcK	ybcK	P77698 [194–202]	[R].VKTIELIFK.[L]	1319.85	660.42	+2	75.20	13	212.92	81.25%	
prophage integrase IntZ	intZ	P76542 [174–180]	[R].RIGEIFK.[F]	1320.84	660.92	+2	76.86	6	185.15	37.50%	
lipoprotein NlpI	nlpI	P0AFB1 [7–26]	[R].WCFVATALTLAGCSNTSWRK.[S]	2673.40	891.81	+3	78.40	1	180.24	6.25%	
HTH-type transcriptional regulator AscG	ascG	P24242 [306–324]	[R].LIFMLDGGDFSPPKTFSGK.[L]	2744.53	915.51	+3	85.46	1	219.47	6.25%	
50S ribomal protein L31 type B	ykgM	P0A7N1 [11–30]	[R].TVVFHDTSVDEYFKIGSTIK.[T]	2744.49	686.88	+4	85.49	1	194.53	6.25%	
tRNA (guanine-N(1)-)-methyltransferase	trmD	P0A873 [69–75]	[R].DAIHAAK.[A]	1183.72	395.25	+3	31.52	2	214.27	12.50%	
ATP-dependent RNA helicase HrpA	hrpA	P43329 [988–993]	[R].SLQDLK.[D]	1162.71	581.86	+2	59.70	2	221.69	12.50%	
Transcriptional repressor PifC	pifC	P10030 [2–8]	[M].LSQLNLR.[F]	1072.67	536.84	+2	54.13	1	195.86	6.25%	
histidinol-phphate aminotransferase	hisC	P06986 [170–178]	[R].TLLELTRGK.[A]	1488.95	744.97	+2	82.06	1	181.64	31.25%	945
Probable fructelysine utilization operon transcriptional repressor	frlR	P45544 [179–197]	[R].VVSDKKTIDIFAATRPQAK.[W]	2318.33	773.44	+3	86.67	5	218.51	6.25%	
DNA polymerase III subunit delta	holA	P28630 [293–299]	[R].LSQTQLR.[Q]	1075.63	538.32	+2	46.97	1	186.37	6.25%	
glutamine-binding periplasmic protein	gln *H*	P0AEQ3 [222–227]	[K].VNGALK.[T]	831.51	416.26	+2	28.16	3	206.69	18.75%	135
Kojibie phphorylase	ycjT	P77154 [555–573]	[K].QTILLDYSRAEVNEMQILK.[Q]	2739.50	685.63	+4	82.89	1	215.42	6.25%	
glyceraldehyde-3-phphate dehydrogenase A	gapA	P0A9B2 [218–225]	[K].VLPELNGK.[L]	1328.82	664.92	+2	69.48	3	252.73	18.75%	1
uncharacterized protein YgbA	ygbA	P25728 [45–58]	[R].LDKCVFGEEKPACK.[Q]	1795.93	599.31	+3	66.34	2	195.97	12.50%	
50S ribomal protein L7/L12	rplL	P0A7K2 [110–121]	[K].ALEEAGAEVEVK.[-]	1702.96	568.33	+3	63.17	9	252.42	56.25%	21
translation initiation factor IF-2	infB	P0A705 [1–7]	[-].MTDVTIK.[T]	1036.59	518.80	+2	71.62	2	201.38	12.50%	48
putative hydrolase YuaR	yuaR	P34211 [159–164]	[K].QLILQK.[I]	1200.81	600.91	+2	66.25	1	185.42	6.25%	
nickel import ATP-binding protein NikE	nikE	P33594 [125–136]	[K].SEQLARASEMLK.[A]	1591.87	531.29	+3	71.02	1	195.59	6.25%	
antiadapter protein IraM	iraM	P75987 [92–100]	[K].CLHNSCIIK.[I]	1259.68	630.34	+2	84.12	1	191.4	6.25%	
major outer membrane lipoprotein Lpp	lpp	P69776 [53–59]	[R].SDVQAAK.[D]	1176.70	588.85	+2	33.77	1	190.35	6.25%	466
RecBCD enzyme subunit RecC	recC	P07648 [688–706]	[R].QLAPLGFDLMSQKPKRGDR.[S]	2403.30	801.78	+3	85.54	1	180.46	6.25%	
Tol-Pal system protein TolA	tolA	P19934 [118–125]	[K].KQAEEAAK.[Q]	1333.77	667.39	+2	45.17	1	189.61	6.25%	1001
*N*-acetylneuraminate lyase	nanA	P0A6L4 [242–248]	[K].VIDLLIK.[T]	1271.87	636.44	+2	86.04	1	205.75	6.25%	
Tat proofreading chaperone DmsD	dmsD	P69853 [95–113]	[R].ESVLFGDSTLALRQWMREK.[G]	2725.47	909.17	+3	77.51	1	183.75	6.25%	960
Inner membrane protein YiaV	yiaV	P37683 [163–168]	[R].DIDVAR.[Q]	917.53	459.27	+2	41.97	1	206.14	6.25%	
xyle transport system permease protein XylH	xylH	P0AGI4 [268–279]	[R].IYAIGGNLEAAR.[L]	1477.82	739.41	+2	61.15	1	190.99	6.25%	
uncharacterized protein YmfJ	ymfJ	P75973 [2–7]	[M].NEQNLK.[H]	974.55	487.77	+2	55.13	1	184.83	6.25%	
putative DNA repair helicase RadD	radD	P33919 [253–266]	[R].KGVMIFAATVEHAK.[E]	1960.15	654.05	+3	91.65	1	193.55	6.25%	
DNA base-flipping protein	atl	P0AFP2 [67–73]	[R].QVGGVLK.[R]	930.58	465.80	+2	51.98	1	189.08	6.25%	
elongation factor Tu 2	tufB	P0CE48 [239–249]	[K].VGEEVEIVGIK.[E]	1629.98	544.00	+3	74.62	2	208.23	12.50%	11
HTH-type transcriptional regulator McbR	mcbR	P76114 [81–87]	[R].QLDEINR.[I]	1117.61	559.31	+2	49.29	1	197.73	6.25%	
probable bifunctional chitinase/lysozyme	chiA	P13656 [851–862]	[R].NLGMVGIWSIAR.[D]	1562.86	521.62	+3	63.63	1	181.44	6.25%	
chaperone protein DnaK	dnaK	P0A6Y8 [415–421]	[K].NTTIPTK.[H]	1003.60	502.30	+2	35.14	1	198.32	6.25%	15
anaerobic sulfatase-maturating enzyme homologue YdeM	ydeM	P76134 [294–308]	[K].SELKTMNSVQLTAQK.[K]	1909.01	637.00	+3	76.73	1	190.14	6.25%	
regulator of sigma-E protease RseP	rseP	P0AEH1 [320–339]	[R].QYGPFNAIVEATDKTWQLMK.[L]	2586.31	862.78	+3	83.12	1	187.65	6.25%	
4-hydroxybenzoate octaprenyltransferase	ubiA	P0AGK1 [61–79]	[R].AAGCVVNDYADRKFDGHVK.[R]	2294.15	765.39	+3	66.52	1	194.66	6.25%	
*ascorbate-specific PTS system EIIA component*	*ulaC*	*P69820[3–11]*	*[K].LRDSLAENK.[S]*	*1045.56*	*523.29*	*+2*	*50.41*	*1*	*215.55*	*0.00%*	
*aspartate aminotransferase*	*aspC*	*P00509[333–343]*	*[K].GANRDFSFIIK.[Q]*	*1267.68*	*634.34*	*+2*	*69.17*	*1*	*222.59*	*0.00%*	*29*
*ATP synthase subunit alpha*	*atpA*	*P0ABB0[165–175]*	*[R].ELIIGDRQTGK.[T]*	*1229.68*	*615.35*	*+2*	*30.30*	*1*	*228.28*	*0.00%*	*33*
*biotin carboxylase*	*accC*	*P24182[315–324]*	*[R].IAAGQPLSIK.[Q]*	*998.59*	*499.80*	*+2*	*49.58*	*1*	*181.48*	*0.00%*	*120*
*chaperone protein DnaK*	*dnaK*	*P0A6Y8[167–183]*	*[K].RIINEPTAAALAYGLDK.[G]*	*1816.00*	*606.01*	*+3*	*64.08*	*1*	*285.59*	*0.00%*	*15*
*chromomal replication initiator protein DnaA*	*dnaA*	*P03004[310–316]*	*[K].ADENDIR.[L]*	*1061.54*	*531.27*	*+2*	*16.65*	*1*	*194.01*	*0.00%*	
*DNA polymerase III subunit delta*	*holA*	*P28630[11–18]*	*[R].AQLNEGLR.[A]*	*901.47*	*451.24*	*+2*	*30.19*	*1*	*190.46*	*0.00%*	
*enolase*	*eno*	*P0A6P9[406–411]*	*[K].YNQLIR.[I]*	*806.45*	*403.73*	*+2*	*30.76*	*1*	*186.21*	*0.00%*	*7*
*excinuclease cho*	*cho*	*P76213[101–111]*	*[K].EQQPLFNKRLR.[R]*	*1429.79*	*715.40*	*+2*	*58.57*	*1*	*194.27*	*0.00%*	
*glyceraldehyde-3-phphate* dehydrogenase A	*gapA*	*P0A9B2[214–225]*	*[K].AVGKVLPELNGK.[L]*	*1224.73*	*408.92*	*+3*	*39.82*	*1*	*191.31*	*0.00%*	*1*
*glyceraldehyde-3-phphate* dehydrogenase A	gapA	*P0A9B2[226–232]*	*[K].LTGMAFR.[V]*	*795.42*	*398.22*	*+2*	*43.10*	*1*	*179.87*	*0.00%*	*1*
*glyceraldehyde-3-phphate* dehydrogenase A	*gapA*	*P0A9B2[218–232]*	*[K].VLPELNGKLTGMAFR.[V]*	*1645.91*	*549.31*	*+3*	*68.35*	*1*	*191.49*	*0.00%*	*1*
*glyceraldehyde-3-phphate* dehydrogenase A	*gapA*	*P0A9B2[218–232]*	*[K].VLPELNGKLTGMAFR.[V]*	*1661.90*	*554.64*	*+3*	*60.08*	*1*	*395.37*	*0.00%*	*1*
*glyceraldehyde-3-phphate* dehydrogenase A	*gapA*	*P0A9B2[218–232]*	*[K].VLPELNGKLTGMAFR.[V]*	*1662.89*	*554.97*	*+3*	*63.85*	*1*	*237.95*	*0.00%*	*1*
*high-affinity zinc uptake system protein ZnuA*	*znuA*	*P39172[275–286]*	*[R].MGTLDPLGTNIK.[L]*	*1259.67*	*630.34*	*+2*	*84.48*	*1*	*208.47*	*0.00%*	*591*
*isoleucine-tRNA ligase*	*ileS*	*P00956[432–439]*	*[R].AQSLKEIK.[G]*	*917.53*	*459.27*	*+2*	*85.49*	*4*	*191.84*	*0.00%*	*72*
*low-affinity inorganic phphate transporter PitA*	*pitA*	*P0AFJ7[332–337]*	*[K].LSLDQR.[S]*	*732.39*	*366.70*	*+2*	*29.73*	*1*	*183.61*	*0.00%*	
*nitric oxide reductase FlRd-NAD(+) reductase*	*norW*	*P37596[365–373]*	*[R].MKEAFGLLK.[T]*	*1036.59*	*518.80*	*+2*	*71.51*	*3*	*215.1*	*0.00%*	
*probable acyltransferase YihG*	*yihG*	*P32129[119–128]*	*[K].HIPMNKYFLK.[Q]*	*1291.69*	*646.35*	*+2*	*75.18*	*1*	*183.54*	*0.00%*	
*protein YehF*	*yehF*	*P33345[31–40]*	*[K].VGTKGQSQIK.[S]*	*1045.60*	*523.30*	*+2*	*84.28*	*1*	*203.12*	*0.00%*	
*protein YqfH*	*yqfH*	*P0DPP4[2–9]*	*[M].INQVSVYR.[Q]*	*980.50*	*490.76*	*+2*	*52.30*	*1*	*187.12*	*0.00%*	
*thiamine kinase*	*thiK*	*P75948[5–11]*	*[R].SNNPITR.[D]*	*803.39*	*402.19*	*+2*	*19.64*	*1*	*184.47*	*0.00%*	
*Tol-Pal system protein TolA*	*tolA*	*P19934[103–108]*	*[R].LKQLEK.[E]*	*758.48*	*379.74*	*+2*	*17.05*	*1*	*184.2*	*0.00%*	*1001*
*transaldolase A*	*talA*	*P0A867[92–99]*	*[R].VSTEVDAR.[L]*	*876.44*	*438.73*	*+2*	*19.09*	*2*	*246.62*	*0.00%*	*976*
*uncharacterized protein YdaV*	*ydaV*	*P77546[180–197]*	*[K].NEQVVLHQIVDRRTASMR.[S]*	*2170.10*	*724.04*	*+3*	*82.18*	*2*	*190.12*	*0.00%*	
*uncharacterized protein YdaV*	*ydaV*	*P77546[180–197]*	*[K].NEQVVLHQIVDRRTASMR.[S]*	*2171.09*	*724.37*	*+3*	*82.16*	*2*	*186.46*	*0.00%*	
*uncharacterized protein YdbH*	*ydbH*	*P52645[370–384]*	*[R].SKGRVIDSLDIDEIR.[W]*	*1715.93*	*572.65*	*+3*	*86.03*	*1*	*186.08*	*0.00%*	
*uncharacterized protein YegR*	*yegR*	*P76406[81–87]*	*[K].MQIVELK.[T]*	*860.49*	*430.75*	*+2*	*59.24*	*1*	*184.26*	*0.00%*	
*uncharacterized protein YicN*	*yicN*	*P0ADL3[27–34]*	*[K].AIRRLSDR.[L]*	*986.59*	*493.80*	*+2*	*52.33*	*1*	*275.88*	*0.00%*	

a Italic = observation only without quantification.

An alternative FDR ≤ 0.05 control was performed with the Target Decoy PSM Validator node in Proteome Discoverer. This approach resulted in 79 PSMs yielding 69 peptides and 60 proteins, with most proteins (16 and 55, respectively) identified by one peptide. Out of these, 12 proteins were the same. Out of the five proteins discovered with ≥2 peptides (Tables 1 and 2), glyceraldehyde-3 phosphate dehydrogenase 3 A with ≥2 peptides was the same in both approaches to FDR control.

An additional FDR control was the peptide abundance rank among all peptides quantified in the bulk analysis. The assumption was made that any peptide detected in single and double cells should be among the top 25% of most abundant peptides in bulk analysis. Yet another FDR control was implemented in the form of the 25% threshold for channel occupancy, which is the percentage of TMT channels among the 96 single and double cells for which nonzero abundance was detected.

With a ≤ 25% rank cutoff for the peptide abundance in bulk analysis and a ≥ 25% channel occupancy, 32 peptides belonging to 12 proteins were detected that passed the FDR ≤ 0.05 threshold in either of the above approaches (Tables 1 and 2). Among these proteins, one ribosomal protein (50S ribosomal protein L7/L12, P0A7K2) was readily detected by both methods as well as translation initiation factor (P0A705), that is a key component of protein synthesis, indirectly related to the ribosomal complex.

**Figure 3:**
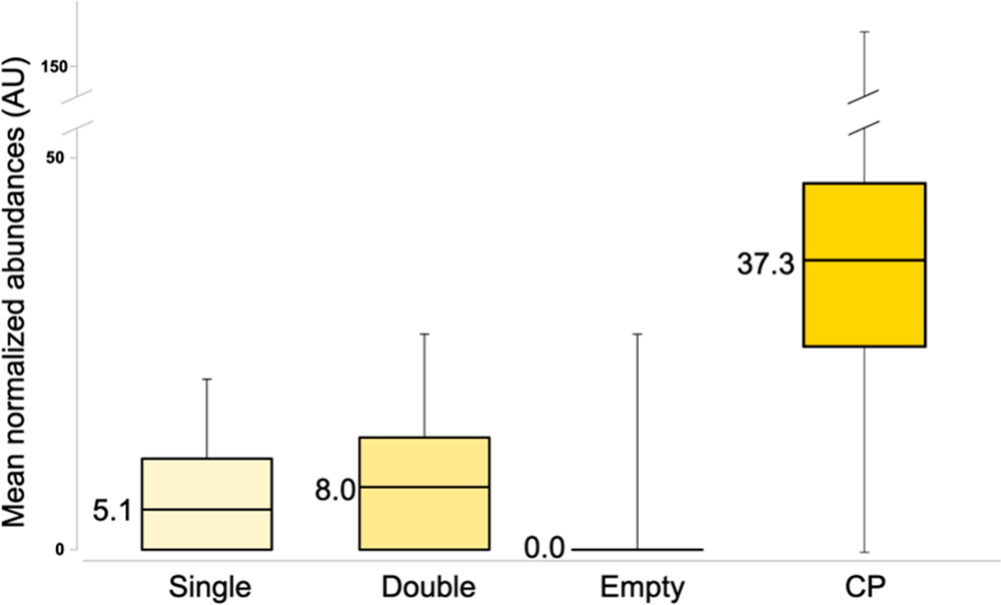
Distributions of the mean abundances calculated with eight bacterial proteins quantified across the data set. The median values are provided for easier comparison.

The distribution of the relative abundances of the TMT reporter ion for the above peptides in the single- and double cell channels is shown in Figure 3. The mean normalized abundances of eight quantified proteins (glyceraldehyde-3-phosphate dehydrogenase A, 2-iminobutanoate/2-iminopropanoate deaminase, 50S ribosomal protein L7/L12, elongation factor Tu 2, biotin carboxylase detected by the Percolator method and glutamine-binding periplasmic protein, translation initiation factor IF-2, chaperone protein DnaK detected by the Targeted Decoy method) were used together to compare the levels of these proteins in samples with single and double cells. For comparison, the data for the channels corresponding to CP with 250 cells as well as for empty channels are also provided. The average value in the latter was statistically indistinguishable from zero, which indicates the absence of carry-over. The average abundance in the double bacteria channels was (60 ± 10)% higher than that in the single bacterium channels, largely consistent with our expectations. As expected, the average abundance in the CP channels was much higher, even though the difference by a factor of 7 with the single bacterium channels was much below the theoretical value of 250. This discrepancy can be attributed to the compression effect in isobaric labeling,^24^ as some unlabeled peptides with zero abundances in all TMT reporter channels were also present in the analyzed samples.

## Conclusions

Summarizing, we have shown that a slightly modified SCoPE MS approach is powerful enough to detect at least a dozen proteins from single bacteria and differentiate quantitatively between one and two bacteria. To the best of our knowledge, this is the first report on MS-based proteomics of single bacterium cells.
